# Soluble endothelial protein C receptor (sEPCR) is likely a biomarker of cancer-associated hypercoagulability in human hematologic malignancies

**DOI:** 10.1002/cam4.11

**Published:** 2012-07-23

**Authors:** Elodie Ducros, Shah Soltan Mirshahi, Anne-Marie Faussat, Pezhman Mirshahi, Sophie Dimicoli, Ruoping Tang, Julia Pardo, Jdid Ibrahim, Jean-Pierre Marie, Amu Therwath, Jeannette Soria, Massoud Mirshahi

**Affiliations:** 1 INSERM, UMRS 872, CRC, Université Pierre et Marie Curie-Paris 6, Université Paris DescartesParis, France; 2 Stago R&DGenevilliers, France; 3 UMRS 938, Université Pierre et Marie Curie-Paris 6Paris, France; 4 Tumor Bank « Leukemia », Hôpital Saint-Antoine, Assistance-Publique-Hôpitaux de ParisParis, France; 5 Département d'hématologie, Hôpital Saint-Antoine, Assistance-Publique Hôpitaux de ParisParis, France

**Keywords:** D-dimer, EPCR (CD201), leukemia, protein C, thrombosis

## Abstract

Elevated plasma level of soluble endothelial protein C receptor (sEPCR) may be an indicator of thrombotic risk. The present study aims to correlate leukemia-associated hypercoagulability to high level plasma sEPCR and proposes its measurement in routine clinical practice. EPCR expressions in leukemic cell lines were determined by flow cytometry, immunocytochemistry, and reverse transcription polymerase chain reaction (RT-PCR). EPCR gene sequence of a candidate cell line HL-60 was also determined. Plasma samples (*n* = 76) and bone marrow aspirates (*n* = 72) from 148 patients with hematologic malignancies and 101 healthy volunteers were analyzed by enzyme-linked immunosorbent assay (ELISA) via a retrospective study for sEPCR and D-dimer. All leukemic cell lines were found to express EPCR. Also, HL-60 EPCR gene sequence showed extensive similarities with the endothelial reference gene. All single nucleotide polymorphisms (SNPs) originally described and some new SNPs were revealed in the promoter and intronic regions. Among these patients 67% had plasma sEPCR level higher than the controls (100 ± 28 ng/mL), wherein 16.3% patients had experienced a previous thrombotic event. These patients were divided into: group-1 (*n* = 45) with amount of plasmatic sEPCR below 100 ng/mL, group-2 (*n* = 45) where the concentration of sEPCR was between 100 and 200, and group-3 (*n* = 20) higher than 200 ng/mL. The numbers of thrombotic incidence recorded in each group were four, six, and eight, respectively. These results reveal that EPCR is expressed not only by a wide range of human malignant hematological cells but also the detection of plasma sEPCR levels provides a powerful insight into thrombotic risk assessment in cancer patients, especially when it surpasses 200 ng/mL.

## Introduction

Thrombosis is a common complication in hematological malignancies and solid tumors. The clinical course of cancer is characterized by a thrombophilic state, manifested by venous thromboembolism and microcirculatory disturbances. Solid tumors and leukemias share many thrombogenic factors; however, the incidence of thrombotic complications is higher in malignant hemopathies [1–3] than in solid tumors. Additional prothrombotic factors, such as hyperleukocytosis, increased expression of tissue factor, and excessive fibrinolysis may be responsible 2008a. Acquired activated protein C (APC) resistance may be another mechanism responsible for such hypercoagulation [5].

Unactivated protein C (PC) binds to its specific receptor expressed on endothelial cells (endothelial protein C receptor, EPCR, CD201) which triggers its activation via the thrombin–thrombomodulin complex [5]. APC, together with protein S, degrades factors Va and VIIIa provoking an inhibition of thrombin generation [6–8]. EPCR can be released in plasma by proteolytic cleavage in a free soluble form (sEPCR) [9]. Like the membrane-associated form, the free soluble form binds PC and APC with similar affinities. However, sEPCR has the ability to trap free APC, interfering thereby in its anticoagulant activity, and abrogating its ability to inactivate factor Va [10].

There is currently a renewed interest in EPCR/PC [11, 12]. While EPCR has been detected in solid tumors, very few works report its expression by malignant hematological cells. A specific binding site for APC on human monoblastic leukemia U937 cell responsible for regulation of tissue factor expression has been identified [13]. Cancer cell surface expression of EPCR is related to enhanced cell survival and invasion [14].

The present study emphasizes the significance of sEPCR-EPCR/PC in the hypercoagulability observed in hematological malignancies and its potential prognostic and treatment implications in patient care.

## Materials and Methods

### Antibodies and primers

Primary antibody AF2245 against the EPCR (R&D Systems, Lille, France); biotinylated anti-goat IgG, streptavidin-fluorescein conjugate (Amersham, UK); human recombinant APC (Lilly, France); sEPCR and D-Di asserachrom (Stago, France). The PCR primers were designed with Primer 3 program, BLAST verified, and synthesized by Eurobio (Les Ulis, France).

### Cell lines

Six human leukemia cell lines from ATCC-LGC standard partnership, and one nonleukemic cell line (HMEC-1) from Lonza Basel, Switzerland, used were HL-60, (acute promyelocytic leukemia ATCC,CCL-240); THP-1 (acute monocytic leukemia ATCC, TIB 202) and U937 (histiocytic lymphoma ATCC, CRL-1593.2), K-562 (chronic myelogenous leukemia ATCC, CCL 243); CEM/C1 (acute lymphoblastic leukemia ATCC, CRL 2265), RAJI (Burkitt's lymphoma ATCC, CCL-86), and human microvascular endothelial cell line (HMEC-1 Human Dermal Microvascular Endothelial Cells, Lonza, CC-2543). Cultures were at 37°C in a moist atmosphere containing 5% CO_2_ in RPMI-1640 medium with antibiotics and with or without 10% fetal calf serum. Serum-free cell culture were harvested after an overnight incubation and frozen at −20°C for further analysis.

### Plasma samples and bone marrow aspirates

Blood (*n* = 76) and bone marrow (*n* = 72) samples from patients with hematological malignancies were obtained from the Hôtel-Dieu Hospital (Paris) after informed consent. As plasmatic and bone marrow sampling is part of the routine management of patients, only oral consent was needed. A total of 148 patients (86 men and 62 women) with age range 39–90 years (mean ± SD: 62 ± 14 years) were selected. The evolution of the disease varied between 0 (newly diagnosed) and 10 years. Diagnosis was established according to international criteria. The cases included were acute myeloid leukemia (AML, *n* = 66), chronic myeloid leukemia (CML, *n* = 12), acute lymphoblastic leukemia (ALL, *n* = 8), chronic lymphoblastic leukemia (CLL, *n* = 25), lymphoma (*n* = 15), multiple myeloma (*n* = 11), and unclassified (*n* = 11). Blood from healthy volunteers was obtained from the French National Blood Bank. Blood was collected on sodium citrate and plasma was obtained after 30 min centrifugation (3000 rpm). The bone marrows collected by medullar aspiration were centrifuged (1500 rpm) for 10 min. Plasmas and bone marrow supernatants were frozen at –20°C until further analyses.

### Detection of EPCR in cell lines

Was performed by immunocytochemistry as described [15]: Briefly, the cells were fixed using a mixture containing paraformaldehyde (3% for fixation) and triton (1% for permeabilization) and then incubated for 1 h at 4°C with either 20 μg/mL EPCR specific antibodies or isotype-matched control mAb. Cells were then incubated successively with biotinylated secondary antibody and streptavidin–fluorescein, and analyzed using flow cytometry on a FACScan.

### Evaluation of sEPCR and D-dimer

sEPCR and D-dimer levels were measured using ELISA in patients samples and in cultured cell supernatants using Asserachrom EPCR and D-dimer, respectively. Plasma and bone marrow supernatants were diluted 1:51 as recommended by the supplier, whereas cell supernatants were used undiluted.

### RT-PCR analysis

RNA was prepared using the Nucleospin RNA-II kit (Macherey-Nagel, France). Mu-MLV reverse transcriptase and oligo(dT) primers for reverse transcription and Taq DNA polymerase for polymerase chain reaction (PCR) were from Gibco, France. Specific primers for EPCR synthesized were as follows: sense: 5′-CAA CTT CAG GAT GTT GAC AA-3′; antisense: 5′-CTA CAG CCA CAC CAG CAA T-3′ to yield a product size of 692 bp [16]. Negative control was performed with water and positive control with beta-2 microglobulin primers (sense: 5′-CAT CCA GCG TAC TCC AAA GA-3′; antisense: 5′-GAC AAG TCT GAA TGC TCC AC-3′). The PCR products, along with a 100 bp DNA ladder, were analyzed by electrophoresis on agarose gels containing ethidium bromide.

### Gene sequencing

The HL-60 RNA extract was subjected to reverse transcription. HL-60 DNA was prepared using the Nucleospin Tissue kit (Macherey-Nagel France). Genomic DNA and complementary DNA (cDNA) samples were sequenced by Qiagen. Samples were amplified with HotStar-Taq Plus DNA Polymerase using custom-made primers. These primers were selected according to EPCR sequence available under GenBank accession number AF106202 or BC01445. Reference AF106202 includes the entire gene and promoter region (8167 bp), whereas BC01445 represents the mRNA (1381 bp). Both DNA strands were sequenced with BigDye 3.1 Terminator Chemistry (Applied Biosystems, USA) using ABI Sequence Analyzer 3730XL. The genomic DNA sequence was aligned with the GenBank sequence AF106202, whereas the cDNA sequence was aligned with BC01445.

## Results

### Leukemic cell lines express EPCR

**Figure 1:**
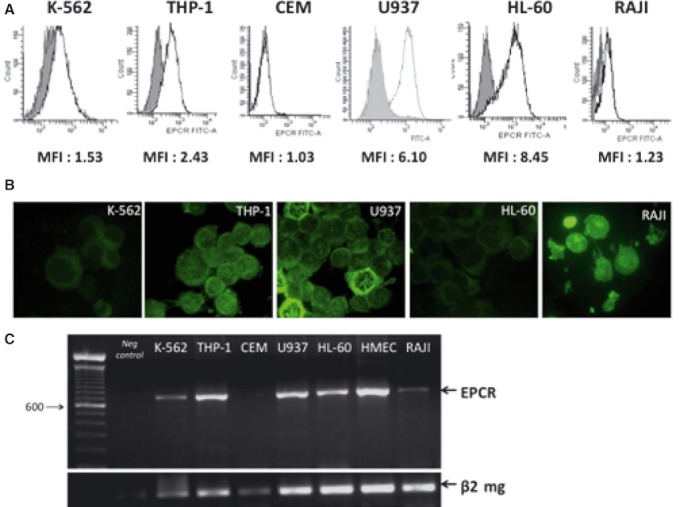
EPCR expression by leukemic cell lines. (A) EPCR flow cytometry analysis. Fluorescence-activated cell sorting (FACS) gate: this graph shows the number of cells (X-axis) and the level of fluorescence emitted (Y-axis) by the labeled cells. The solid line shows EPCR staining; the shaded areas represent isotype controls. The mean fluorescent intensity (isotype/EPCR-specific MFI ratio) is indicated underneath the FACS gate. (B) EPCR immunocytochemistry. Leukemic cells were spotted on microscope slides by centrifugation, fixed and successively incubated with EPCR antibody, appropriate biotinylated secondary antibody, and fluoresceine iso thio cyanate. Isotypic control was performed in parallel. Initial magnification is ×400. (C) EPCR amplification. Gel electrophoresis of RT-PCR products. The positive control (endothelial cell line HMEC) gave an amplified band of similar size of 692 bp as observed for K562, THP-1, U937, HL-60, and Raji. The control without nucleic acid (Neg control) remained negative, whereas the control performed with beta2-microglobulin primers was positive for all samples.

(a)Membrane-bound EPCR protein was identified by fluorescence-activated cell sorting (FACS) in the five cell lines. Significant fluorescence intensities mean fluorescence intensity (MFI), corrected for values in related controls, was seen (Fig. 1A). Immunofluorescence studies (Fig. 1B) confirmed the results obtained by immunocytochemistry. Among the six cell lines tested we found that the MFI ratios were the highest for HL-60 and THP-1 cells and lowest for K-562, whereas the CEM cells were negative.

(b)The supernatants from the six leukemic cell lines were also tested by ELISA for the presence of sEPCR. Only the U937 cells released sEPCR in the culture medium. In all, 10^6^ U937 cells shed about 450 ng of soluble EPCR, whereas sEPCR was undetected in supernatants from the other four cell lines.

(c)These cells (five of the six leukemic cell lines) express an RNA transcript, which when amplified by RT-PCR gave a clean band of 692bp which is consistent with the predicted size for EPCR specific transcripts. The CEM cells (acute lymphoid leukemic cells) lacked this band. The cell line HMEC served as reference for identification of the transcript size (Fig. 1C).

### Primary sequences of EPCR DNA as well as cDNA of HL-60 cells

**Figure 2:**
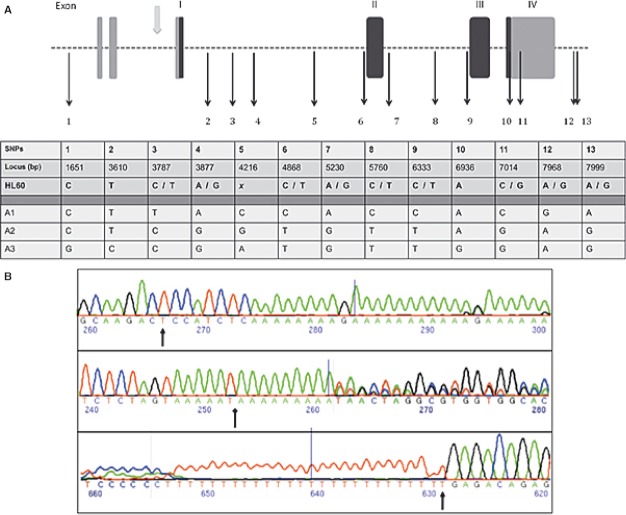
EPCR gene sequence analysis from human acute myelomonocytic leukemic cell line HL-60. Leukemic HL-60 cells EPCR DNA alignment with GenBank reference AF106202. DNA extracts were shipped to Qiagen Sequencing Service. Both DNA strands were sequenced and nucleotides were numbered according to the appropriate GenBank reference. (A) The 13 single nucleotide polymorphisms (SNPs) initially described in endothelial cells were also found here allowing us to designate three haplotypes (A1, A2, and A3). Exons are presented as vertical rectangular blocks; darkly shaded regions are the ones that are transcribed. Black arrows indicate the SNPs. Gray arrow shows a promoter region. (B) Chromatograms show additional polymorphisms detected in HL-60. The upper panel shows the thymidine insertion (locus 260), the middle one the adenosine deletion (locus 840), and the lower panel points out the thymidine amplification (locus 7650). On chromatograms, the nucleotides are arbitrarily numbered. X in the table denotes unconfirmed nucleotides.

One cell line, namely HL-60, was selected for verifying the integrity in these leukemic cells of the gene coding for EPCR. The locus corresponding to EPCR gene from HL-60 cells was sequenced and compared with a consensus sequence of the endothelial cell gene: genomic DNA of HL-60 cell line was sequenced and was compared with BC01445 (1381 bp) and AF106202 (8167 bp) Gene Bank loci, respectively. The results presented in Figure 2A shows the conserved identity, including the 13 single nucleotide polymorphisms (SNPs), reported earlier in the endothelial gene [16]. However, within the 5′UTR region, a thymidine insertion (locus 260) and an adenosine deletion (locus 840) were found in the HL-60 gene (Fig. 2B). Moreover, thymidine amplification was detected within exon IV, in an untranslated region (locus 7650). These changes, being in the untranslated region do not affect the protein sequence. HL-60 cells carry both C and T at nucleotide position 3787 and A and G at nucleotide position 5230 on the genomic DNA sequence. This characterizes heterozygosis for A1 and A2 haplotypes (Fig. 2B). The EPCR cDNA sequencing and alignment with endothelial gene reference sequence confirmed the HL-60 A1/A2 genotype (results not shown). The data from the various approaches confirm that EPCR of leukemic cells, even after numerous passages and in vitro life is in essence identical to the endothelial cell reference gene.

### Retrospective clinical study

**Figure 3:**
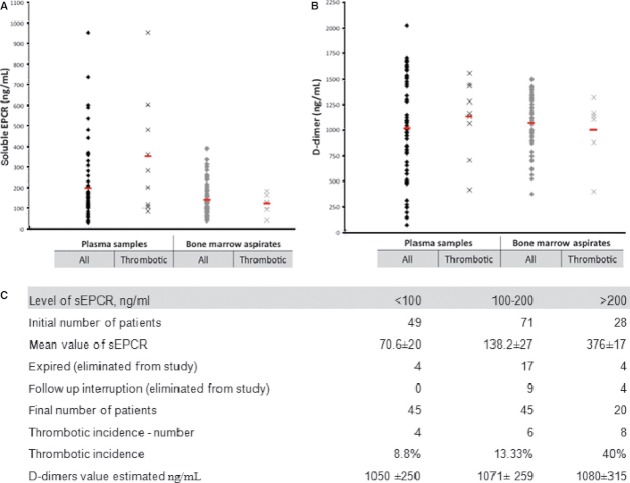
Soluble EPCR and D-dimer quantification in plasma and medullar samples of leukemic patients. Plasma and medullar samples of leukemic patients were collected and subjected to ELISA quantification of either soluble EPCR (A) or D-dimer (B). Darkly shaded blocks represent all patients, whereas crosses show thrombotic patients. Mean concentrations of sEPCR and D-dimer are represented by a red dash. Results are representative of three independent experiments, each of which gave similar results. As presented in Figure 3C, the initial number of our study was 148 patients. Some of them were expired (*n* = 25) or eliminated from study because of follow-up interruption (*n* = 13). Final number of patients (110 patients) divided into three sub-groups: group-1, -2, and -3. The level of sEPCR and thrombotic incidence for each groups described.

In a retrospective study (Fig. 3) that focused on sEPCR, we observed an overall increase in plasma sEPCR in leukemic patients (196.7 ng/mL; *n* = 76) as compared with healthy controls (100 ± 28 ng/mL; *n* = 101). However, 33% of the leukemia patients had a plasma concentration of sEPCR within the normal range (Fig. 3A). They are patients either in complete remission or under chemotherapy or with bone marrow aplasia. Curiously, the mean value of sEPCR obtained in the supernatant of bone marrow aspirate (*n* = 72) was lower than that observed in patients' plasma. D-dimer levels ranged between 530 and 2030 ng/mL in 90% of the patients, which is higher than the cutoff value of 500 ng/mL for healthy volunteers (result not shown). The D-dimer data, however, corroborates with this retrospective study (Fig. 3B).

The initial number of patients in this study was 148, 25 of these patients expired while 13 more were untraceable and dropped (Fig. 3C), leaving 110 patients in this study. Of this, 59% had the sEPCR level higher than the controls (100 ± 28 ng/mL). Of this, 18 (16.3%) patients had a previous thrombotic event. The patients were divided into three sub-groups (Fig. 3B): group-1 (*n* = 45) with amount of plasmatic sEPCR below 100 ng/mL (mean value of sEPCE was 70, 6 ± 20), group-2 (*n* = 45) the sEPCR was between 100 and 200 (MV = 138, 2 ± 27), and group-3 (*n* = 20) where it was higher than 200 ng/mL (MV = 376 ± 17). The numbers of thrombotic incidence in each group was four (8.8%), six (13%), and eight (40%), respectively. D-dimer values estimated in three groups are the same and are 1050 ± 250, 1071 ± 259, and 1080 ± 315, respectively, the differences not being significant. Among these patients, 74% were AML, CML, ALL, and CLL. They together represented 76.9% of thrombosis events, with plasmatic sEPCR levels indicating less than 100 ng/mL for 25%, between 100 and 200 ng/mL for 35%, and above 200 ng/mL for 40%. AML alone represented 60% of all patients. Nearly two-thirds of these had thrombotic incidences and they could be split into three groups again depending on the plasmatic sEPCR levels.

## Discussion

Thrombotic complications associated with hematologic malignancies represent one of the main causes of mortality. Several hemostatic markers are currently used to predict the advent of thrombosis [3, 17, 18]. However, none of these markers directly indicate the course and progression of the disease.

Here we report, for the first time, the detection of sEPCR in different cell lines originating from malignant hemopathies. One of them (U937) was found to secrete sEPCR into the culture medium. In addition, sequence data obtained from HL-60 (taken as a representative cell line), reflects the integrity of the sECPR gene in these cells. It is now claimed that sEPCR and thrombophilic state have close links particularly in the presence of haplotype-3 [9, 16, 19, 20]. It is possible that the cell line U937 that secrets sEPCR could be homozygous for haplotype-3. Presence of haplotype-3 has been demonstrated in several cell lines of solid tumor origin in recent study emanating from our laboratory [21].

To determine whether sEPCR released from leukemic cells might be an indicator of the coagulation status in patients, we evaluated sEPCR in plasma and in bone marrow supernatants of patients with malignant hemato-logical diseases.

Among the 110 patients with complete follow-up history, 59% had a plasma sEPCR level higher than the controls (100 ± 28 ng/mL) and 18 patients had a previous thrombotic event (16.3%). The numbers of thrombotic incidence in each group increased with sEPCR levels. The thrombotic risk in patients increased when the level of sEPCR was higher than 200 ng/mL, suggesting that sEPCR released from malignant cells could serve as a “trap” for protein C, preventing its binding to EPCR on the surface of endothelial cells.

The association of sEPCR and thrombosis was confirmed for each group of patients. Among these, 76.9% of thrombosis occurred in AML, CML, ALL, and CLL patients. AML alone represents 60% of total thrombosis. It stands out that 41.7% of these AML patients show plasmatic sEPCR above 200 ng/mL. These results confirm the role of sEPCR in thrombotic state observed in hematologic malignancies and particularly in AML.

Quantification of plasma sEPCR, along with other markers such as D-Dimers during treatment or during the course of remission would allow clinicians to discriminate between patients at imminent risk of thromboembolism and those who are not and thereby adopt the required care. Interestingly, in leukemic patients, the levels of sEPCR in the supernatant of bone marrow aspirates were lower than that of plasma levels and were identical whether these patients presented a previous episode of thrombosis or not. Hence the need to focus attention on blood analysis.

In conclusion, determination of plasma sEPCR provides us not only a powerful tool for the assessment of thrombotic risk factor in patients but also arms us for taking preventive measures in time. It should be possible to put together a protocol easily applicable in routine clinical laboratory that includes quantitation of sEPCR in patients with hematological malignancies.
